# Difficult extubation of a damaged neural integrity monitor electromyogram tracheal tube: A case report

**DOI:** 10.1097/MD.0000000000020250

**Published:** 2020-06-19

**Authors:** HoSik Moon, SungJin Hong, ChoonHo Sung, JinYoung Chon, JuEun Kwak, JiYung Lee

**Affiliations:** Department of Anesthesiology and Pain Medicine, College of Medicine, The Catholic University of Korea, Seoul, Republic of Korea.

**Keywords:** electromyogram tracheal tube, extubation, thyroidectomy

## Abstract

**Introduction:**

The purpose of a neural integrity monitor electromyogram (EMG) tracheal tube is to reduce the risk of damage to the recurrent laryngeal nerves. Complications associated with the use of EMG tube are ventilatory failure, tracheal injury, and difficult extubation.

**Patient concerns:**

We encountered a case of difficult extubation of an EMG tube after thyroidectomy and partial tracheal resection in a 73-year-old woman.

**Diagnoses:**

The cuff was torn intraoperatively; but, it was kept inflated to maintain the integrity of the ventilatory circuit. During extubation, the vocal cord blocked the torn hole on the shoulder of the cuff, which subsequently was filled with air, complicating the extubation.

**Interventions:**

We extubated the EMG tube slowly with the help of videolaryngoscopy with a moderate amount of force and re-intubated with a 6.0-mm ID endotracheal tube.

**Outcomes:**

We examined the airway during and after re-intubation using videolaryngoscopy. The findings were normal and no bleeding or laceration was observed. The subsequent recovery and extubation occurred smoothly.

**Conclusions:**

Awareness of the characteristics and types of damage that can occur in an EMG tube is essential. Because it can be difficult to ascertain the type of damage before extubation, communication between the surgeon and anesthesiologist, along with the preparation for emergency airway management are necessary for cases of difficult extubation.

## 1 Introduction

Intraoperative neuromonitoring is a common practice during thyroid surgery and neck dissection to avoid recurrent laryngeal nerve palsy and is reported to significantly reduce the incidence of recurrent laryngeal nerve injuries.^[1]^ The electromyogram (EMG) tube used for neuromonitoring has features different from that of an ordinary endotracheal tube (ETT). It is a type of reinforced tube made from silicone, with 2 pairs of recording electrodes, 3 cm in length, positioned in contact with the vocal folds.^[2,3]^ The complications associated with EMG tubes, such as ventilatory failure,^[4,5]^ tracheal injury,^[6]^ and difficult extubation,^[7,8]^ have been reported. In this case report, we present a case of difficult extubation of EMG tube appeared to be caused by non-deflation of damaged cuff, after thyroidectomy and partial tracheal resection. This unique case may help anesthesiologists understand the properties of an EMG tube cuff. Rotation of the tube for releasing the intracuff air can be considered as an alternative method for extubation.

## 2 Case presentation

A 73-year-old woman (53 kg, 147 cm, and body mass index, 24) was presented to the operating suite for total thyroidectomy for bilateral papillary thyroid cancer. She was diagnosed with thyroid cancer on sonography during a routine medical checkup. A case of bilateral papillary carcinoma of thyroid was confirmed by computed tomography scan and fine-needle aspiration biopsy. Thereafter, she was transferred from the endocrinology department to the otorhinolaryngology department for operation. Examination by an otorhinolaryngologist was indicative of the presence of right vocal cord palsy and metastases. On enhanced thyroid scan, invasion of the right tracheoesophageal groove was observed. Her past medical history included diabetes and hypertension for more than 10 years, and she took an antihypertensive (losartan potassium, 50 mg), medications for diabetes (glimepiride, 2 mg; metformin, HCl 500 mg; alogliptin benzoate, 34 mg; thioctic acid, 600 mg), and cilostazol (200 mg). Her HbA1c value was 6.2% (reference range, 4.0%–6.0%), and her blood sugar levels ranged from 84 to 110 mg/dL preoperatively. She underwent an appendectomy 40 years ago without any anesthetic complications. Her preoperative electrocardiogram showed a first degree atrioventricular block, and echocardiography showed left ventricular hypertrophy and moderate diastolic dysfunction with normal ejection fraction (70%). Her airway was categorized as Mallampati Class 1. Endoscopic view of the larynx, as viewed by an otorhinolaryngologist, was normal.

After monitoring the electrocardiogram, blood pressure, pulse oximetry, and bispectral index (BIS), we preoxygenated the patient thoroughly. After administration of propofol (80 mg), rocuronium (50 mg), and confirmation of appropriate level of BIS for intubation, we tried to intubate the trachea with a 7.0-mm (internal diameter, 7 mm) neural integrity monitor standard reinforced EMG tube under videolaryngoscopic assistance. Because the tube was too thick for the patient's trachea, we successfully intubated her with an ID 6.0 mm EMG tube. Adequate depth of the tube was confirmed by videolaryngoscopy and auscultation. The cuff pressure of the tube was adjusted to 20 cmH_2_O using a manometer. We anesthetized the patient with total intravenous anesthesia using a target-controlled infusion pump (Orchestra with Base Primea, Fresenius Kabi, France) to infuse remifentanil and propofol with BIS and Train of four monitoring. After beginning the operation, we checked for spontaneous recovery from a neuromuscular blockade with a train of four watch, without disturbing the neuromonitoring. Total thyroidectomy, tracheal wedge resection, and end-to-end anastomosis were performed. Intraoperatively, the right-sided recurrent laryngeal nerve attached to the tumor was sacrificed. Furthermore, the right-sided tracheal wedge was resected and anastomosed because of the metastases into the trachea at the level of the 3rd to 5th tracheal rings. The mechanical ventilation was under control without any circuit leakage.

After completion of the surgery, anesthetic infusion was stopped and sugammadex (200 mg) was administered for reversal of the neuromuscular blockade. After confirmation of the patient's recovery, we tried to extubate her trachea after deflation of the tracheal cuff but felt strong resistance after withdrawal of the tube for 2 to 3 centimeters. Deflation was attempted again but the balloon did not shrink completely, and the resistance to extubation persisted. Therefore, a deeper anesthesia was induced with sevoflurane and the surgeon's opinion was sought for the possibility to anchor the tube by stitching it to the tracheal wall. The surgeon expressed the possibility of intraoperative damage to the cuff by sharp scissors during tracheal dissection; however, was strongly against the anchoring of the tube because the suture site was more proximally situated (closer to vocal cord) to the cuff of the tube. A consensus was achieved that the EMG tube was already withdrawn 2 to 3 cm, sufficient to pass through the sutured portion. Endoscopic examination through the tracheal tube or videolaryngoscopy revealed no abnormal findings. For the subsequent attempt at extubation, emergency surgical airway devices along with a videolaryngoscope and an ordinary ETT was kept ready. After the induction of a deeper anesthesia, we tried to extubate the EMG tube slowly under videolaryngoscopic view. With a moderate amount of force, the EMG tube was finally extubated, apparently in a fully inflated state. Thereafter, we intubated the trachea with an ID 6.0-mm ETT under videolaryngoscopic view; the laryngoscopic view was normal without the presence of any bleeding or laceration. Subsequent recovery and extubation were uncomplicated. The patient complained of slight soreness in the throat after recovery. She suffered from dyspnea for several days, caused by an arytenoid swelling seen on fiberoptic laryngoscopy, airway narrowing (1.4 × 0.7 cm), and mild dependent atelectasis in the bilateral lower lobes, which was documented on computed tomography. Finally, she was discharged on the 9th postoperative day after improvement of symptoms.

**Figure 1:**
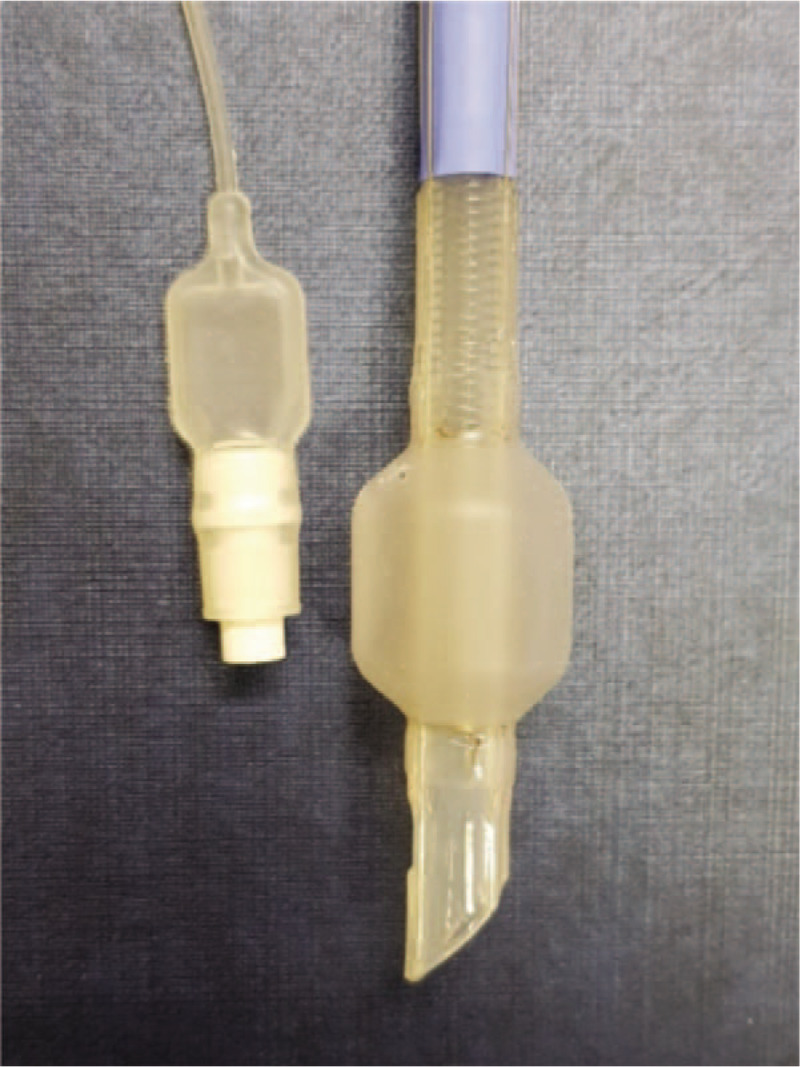
Tear in an electromyogram tube cuff.

**Figure 2:**
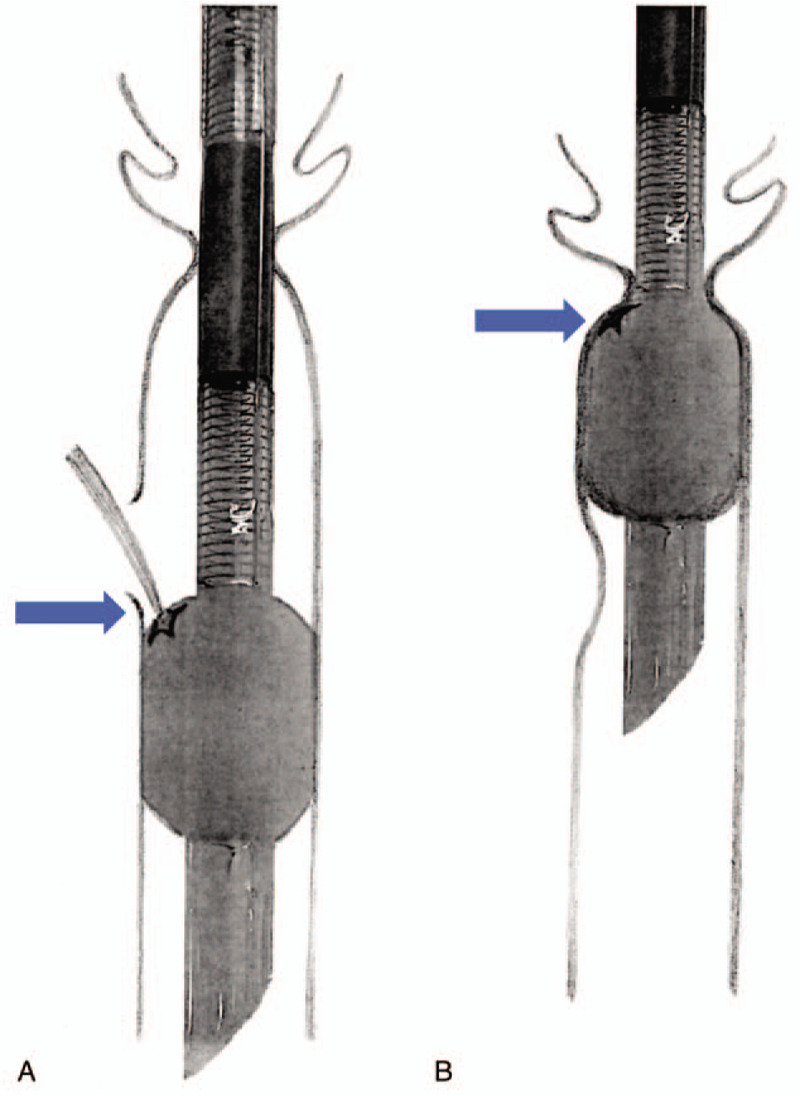
The vocal cord obstructed the torn cuff, so the cuff was not deflated during extubation.

We examined the extubated EMG tube and found a tear in the cuff tear on its right shoulder, which corresponded with the surgical site (Fig. 1). Deflation of the cuff caused it to shrink marginally; however, it expanded spontaneously to its original cylindrical shape. We concluded that the vocal cord obstructed the torn cuff during extubation, preventing the air inside the cuff from escaping (Fig. 2).

This case report was approved by the Institutional Review Board of St. Mary's Hospital, The Catholic University of Korea (approval number SC19ZESE0080). The patient provided written informed consent for publication of this report.

## 3 Discussion

Difficult extubation with regard to “difficult decannulation of the airway” has been rarely reported. It can be caused by mechanical factors related to the patient, surgery, or anesthesia.^[9]^ These mechanical factors include idiopathic subglottic stenosis or severe edema,^[10]^ surgical stitch anchoring the ETT,^[11]^ and incomplete deflation of the ETT cuff^[12]^ either because of ETT cuff malfunction or due to negligence. Although difficult extubation has been reported with various tubes, such difficulties are extremely rare with the use of NIM EMG tubes. It is a type of reinforced tube made of a soft silicone-base with electrodes without the arc-shaped tube curve.

EMG tubes have several characteristics associated with complications. The most serious complication associated with the EMG tube is ventilatory failure.^[4]^ This is caused by cuff over-inflation or herniation. The manufacturer's instructions emphasize on keeping the EMG tube immobile in its inflated state. Furthermore, inefficient ventilation after EMG tube intubation has been identified to occur because of the detachment and perforation of the cuff by an EMG electrode.^[6]^ The difficult extubation of EMG tube has also been reported with oversized tube and separated flap which impedes extubation.^[7,8]^ The outer diameters of EMG tubes are bigger than that of polyvinylchloride (PVC) and reinforced, soft-silicone-based tubes with same ID; therefore, a tube with an adequate size has to be chosen carefully. An excessively large tube, which is in tight contact with the tracheal inner wall can lead to difficult extubation.^[7]^ This might have been the possible cause of difficult extubation experienced in our patient. However, retrospectively, our decision to choose an EMG tube with an ID of 6.0 mm (outer diameters, 8.8 mm) seemed justified. Anesthesiologists have to choose an appropriately sized EMG tube, which is neither too small to induce proper contact of electrodes with the vocal cords and nor too big for smooth intubation and extubation. The use of videolaryngoscope was helpful to confirm the adequate size and depth of the EMG tube.

**Figure 3:**
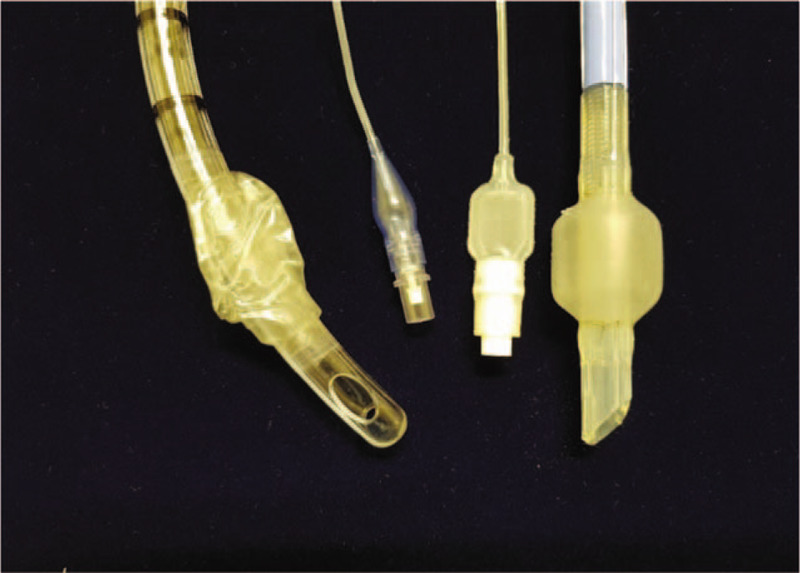
Comparison of the damaged cuffs in a PVC tube and an EMG tube. The cuff of the PVC tube collapses because of an artificial tear; however, the damaged cuff of the EMG tube does not collapse. EMG = electromyogram, PVC = polyvinylchloride.

Unusually, the ventilatory circuit was intact in spite of intraoperative cuff damage in the present case. We suspect 2 possible causes for this observation. First, the elastic force of the silicone cuff may have retained the original contour. After the damage to the EMG tube cuff during tracheal dissection, it kept its own inflated shape rather than collapse. On extubation under videolaryngoscopic view, we observed a round, inflated cuff. We also compared the damaged EMG tube in this report to an artificially damaged PVC tube (Fig. 3). Collapse in the damaged EMG tube cuff was lesser than that in the PVC tube, and moreover, despite damage, it retained its balloon shape. Attempts to deflate the cuff of the damaged tube with cuff tubing led to transient shrinking of the balloon with subsequent spontaneous inflation. The EMG tube cuff is more resistant than the PVC tube cuff. Second possible reason for the intact ventilatory circuit despite cuff damage could be the volume effect exerted by the comparatively thicker silicone cuff in the trachea to maintain ventilator patency.

Anesthesiologists who encounter extubation failure after tracheal surgery near the ETT may suspect that the tube was transfixed because of suturing of the tracheal wall. However, the surgeon in this case excluded that possibility because the suture site was more proximally located to the cuff and the surgeon had confirmed the distal location of the inflated cuff before tracheal closure. Furthermore, we recognized that the tube was already withdrawn 2 or 3 cm from its position before resistance was offered during extubation. The resected trachea was 1.5 × 1.0 × 0.4 cm, and therefore, the tracheal suture area was no more than 2 cm in length. Following withdrawal of the tube for 2 or 3 cm, the cuff seemed to pass through the suture area. Identifying and excluding the possibility of transfixation was difficult even with the use of an endoscope. The clear judgment of a surgeon and communication with anesthesiologists would be important in this aspect.

A valuable clue for cuff damage was that the pilot balloon did not collapse fully and inflated spontaneously against the aspiration of air, which implies damage to the cuff or to the cuff tubing. However, because most tubes collapse spontaneously after cuff damage, we did not anticipate the possibility of an inflated cuff and attempted extubation. The manufacturer recommends complete deflation of the cuff should resistance be encountered during extubation. The self-inflating characteristics of a damaged EMG tube cuff and obstruction of the tear by the vocal cords caused the retention of the intracuff air. After extubation and subsequent examination of the damaged tube, we believe that rotation of the tube at a right angle, clockwise or counterclockwise, might have enabled the easy removal of the torn portion of the cuff from the obstructing vocal cord. This case report has two limitations. The veracity of the theorized mechanisms is yet to be verified. Moreover, a strategy to exclude the possibility of transfixation of the tube to the tracheal wall needs to be identified.

In this case report, we emphasize the characteristics and complications that can occur during the usage of an EMG tube. Although we experienced a difficult extubation of the EMG tube because of cuff damage, this might happen during tracheal surgery with any type of ETT. Clear circumstantial judgement of the operator and anesthesiologists after a frank exchange of views is essential in such cases. Moreover, extubation should be performed with the possibility for surgical airway management.

## Acknowledgments

We would like to thank Editage (www.editage.com) for English language editing and publication support.

## Author contributions

HoSik Moon performed data acquisition and interpretation.

SungJin hong, ChoonHo Sung, JuEun Kwak, and JinYoung Chon were part of the anesthesia team and assisted with drafting of the manuscript.

JiYung Lee drafted and revised the manuscript.
